# Model-Based Reconstructive Elasticity Imaging Using Ultrasound

**DOI:** 10.1155/2007/35830

**Published:** 2007-06-14

**Authors:** Salavat R. Aglyamov, Andrei R. Skovoroda, Hua Xie, Kang Kim, Jonathan M. Rubin, Matthew O'Donnell, Thomas W. Wakefield, Daniel Myers, Stanislav Y. Emelianov

**Affiliations:** ^1^Department of Biomedical Engineering, University of Texas at Austin, Austin, TX 78712, USA; ^2^Institute of Mathematical Problems of Biology, Russian Academy of Sciences, Pushchino, Moscow Region 142290, Russia; ^3^Department of Biomedical Engineering, University of Michigan, Ann Arbor, MI 48109, USA; ^4^Department of Radiology, University of Michigan, Ann Arbor, MI 48109, USA; ^5^Department of Surgery, University of Michigan, Ann Arbor, MI 48109, USA

## Abstract

Elasticity imaging is a reconstructive imaging technique where tissue motion in response to mechanical excitation is measured using modern imaging systems, and the estimated displacements are then used to reconstruct the spatial distribution of Young's modulus. Here we present an ultrasound elasticity imaging method that utilizes the model-based technique for Young's modulus reconstruction. Based on the geometry of the imaged object, only one axial component of the strain tensor is used. The numerical implementation of the method is highly efficient because the reconstruction is based on an analytic solution of the forward elastic problem. The model-based approach is illustrated using two potential clinical applications: differentiation of liver hemangioma and staging of deep venous thrombosis. Overall, these studies demonstrate that model-based reconstructive elasticity imaging can be used in applications where the geometry of the object and the surrounding tissue is somewhat known and certain assumptions about the pathology can be made.

## 1. INTRODUCTION

Elasticity imaging or elastography is a method to remotely estimate elastic properties of biological tissues [1–4]. One of the approaches in elasticity imaging is based on the measurement of local tissue deformation as tissue responds to external or internal quasi-static mechanic loading. Ultrasound, MRI, or other methods can be used to measure the resultant internal tissue motion. Using inverse problem formulations, the elasticity (Young's modulus) distribution is evaluated based on the distribution of the strain tensor components. Initially, elasticity imaging was focused on the noninvasive cancer diagnosis, but then the approach proved to be useful in various other applications including detection of atherosclerotic plaques [5, 6], corneal refractive surgery [7], cardiac strain imaging [8], muscle biomechanics [9], and so forth.

Once the internal tissue motion is measured, the strain image in the tissue can be produced. Usually hard tissue regions are less deformed in comparison with soft regions and, therefore, contrast in strain images is influenced by the tissue elasticity. However, the strain field depends not only on the elastic distribution, but also on global boundary conditions which can be very complicated in real tissues. As a result, the relationship between strain image and elasticity distribution in tissue is not straightforward and reconstruction of Young's modulus is required to determine tissue elasticity quantitatively. Elastic modulus reconstruction in an inhomogeneous material using remote measurements of internal displacements can be posed in a number of ways [10–18]. These approaches can be generally grouped into two categories: direct and indirect (model-based) reconstruction techniques. If all necessary components of the internal displacement vector and strain tensor are available at any point within the object, then reconstruction algorithms based on the mechanical equilibrium equations can be used to describe the unknown distribution of Young's or shear modulus—these techniques, therefore, belong to direct reconstruction methods. Unfortunately, in direct reconstruction methods, it is often difficult to formulate and solve the inverse problem for an arbitrary geometry and elasticity distribution. However, if any prior knowledge or assumptions about the geometry of the object and boundary conditions can be made, the inverse problem can be solved by using repeated solutions of forward problems with adjusted elasticity parameters. Indeed, if elasticity variations of the object within the region of interest can be modeled based on the object geometry or any other assumptions, then a model-based reconstruction can be performed. Therefore, the model-based elasticity imaging methods could be useful in applications where the existence of the pathology is already determined from the imaging study and pathology characterization rather than detection is required. In such cases, the approximate location and geometry of the pathology is known and certain assumption about tissue elasticity distribution can be made. Note here, that both direct and model-based approaches provide information only about relative elasticity distribution. For absolute Young's modulus reconstruction, either reference point or measurements of stress are required.

Ultrasound is widely used in elasticity imaging since motion of the speckle can be tracked over large range of tissue deformations. However, the accuracy of the lateral displacement estimates is less accurate than axial component of the displacement vector. Indeed, for an ultrasound system, the resolution of axial displacement is limited primarily by the frequency bandwidth of the transducer, and the lateral resolution is determined by the width of the ultrasound beam [19, 20]. Since all displacement components and spatial derivatives are needed in direct reconstruction methods, the anisotropy in the accuracy of the displacement vector measurements is an additional source of noise in elasticity images. In contrast, model-based approaches can be formulated using only axial component of the displacement vector to insure that the quality of Young's modulus reconstruction is independent from the quality of lateral motion tracking.

Here we present the model-based elasticity imaging approach illustrated using two potential clinical applications: characterization of liver hemangiomas and differentiation of deep venous thrombi (DVT). For liver hemangioma, the spherical symmetry of the lesion was assumed, and for DVT a blood clot was described as a cylindrically symmetry object. In both cases, the external surface deformations were applied during continuous ultrasound imaging, and the measurements of tissue motion were performed using block-matching cross-correlation technique. Based on the measured strain images, the Young's modulus was reconstructed. These studies demonstrate that model-based reconstructive elasticity imaging can be used in applications where the geometry of the object and the surrounding tissue is assessable and certain assumptions about the pathology can be made from the ultrasound images.

## 2. THEORY

The model-based elasticity reconstruction technique is performed in two successive steps. First, the analytical solution of the forward elastic problem is derived where the displacement and strain fields are determined based on the spatial distribution of Young's modulus in the object and pattern of externally applied deformation. Second, the inverse problem is solved iteratively, where the solution of the forward problem for a modeled object is compared with experimentally measured strains to match the unknown spatial distribution of Young's modulus in a region of interest (ROI). The Young's modulus distribution providing the best agreement is assumed to describe the distribution of elastic properties in the region of interest.

### 2.1. Forward problem

**Figure 1:**
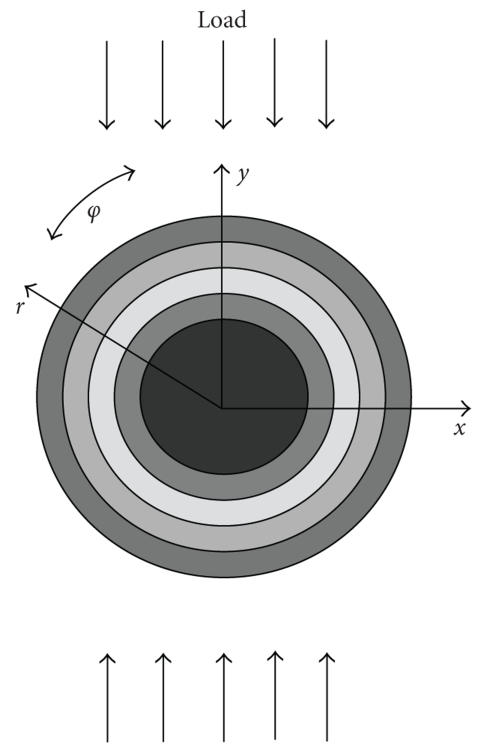
Schematic representation of the deformation model. (*x*, *y*) refer to Cartesian system of coordinates and (*r*, *φ*) refer to either cylindrical or spherical system of coordinates. The inhomogeneity is approximated as a layered round object, where the Young's or shear modulus is a function of only the radial coordinate *r*.

The formulation of a forward problem is based on a uniaxial deformation of an infinite, incompressible medium with spherical or cylindrically shaped inhomogeneities. Here, we consider only incompressible media since most soft tissues are very close to incompressible materials [21]. The forward problem is formulated in a spherical system of coordinates (*r*, *φ*, *θ*) for spherical inclusions and in cylindrical coordinates (*r*, *φ*, *z*) for cylindrical inclusions. The origins of the coordinate systems are placed at the centers of the inhomogeneities. The polar axis of both systems of coordinates is along an applied deformation, that is, an angle *ϕ* is between a radius vector and the deformation direction (see Figure 1). It is assumed that Young's modulus *E*(*r*) is a function of only radial position. To find the solution of forward problem for arbitrary Young's modulus *E*(*r*) over an ROI, we presume that the Young's modulus is constant within each subinterval [*r_i_*, *r*_*i* + 1_], that is, *E*(*r*) : *E_i_* = const, *r* ∈ [*r_i_*, *r*_*i* + 1_], *i* = 1 ⋯ *N*, where *N* is the total number of subintervals covering the region of interest [22]. The displacement vector $\vec{U}$ must satisfy the equations of static equilibrium for isotropic incompressible linear medium on each subinterval [*r_i_*, *r*_*i* + 1_] [23]:

(1) $$\mu \nabla^{2}\vec{U}+\nabla p=0,$$

where *p* is static internal pressure and *μ* is shear elastic modulus. For incompressible media, the shear modulus and Young's modulus are related (*μ* = *E*/*3*), and only one modulus (*μ* or *E*) is sufficient to describe the elastic properties of the material. In addition, the incompressibility condition must be satisfied: div $\vec{U}$ = 0.

Based on Goodier's solution [24, 25], we attempt to find the solution of (1) under the assumption of spherical symmetry (3D case) in the form

(2) $$\begin{array}{c} u_{r}=\frac{1}{4}V_{r}(r)\left[1+3\cos(2\phi )\right],\;\;\;\;\;\;u_{\theta }=0,\\ u_{\phi }=V_{\phi }\left(r\right)\sin\left(2\phi \right),\\ p=P_{0}\left(r\right)+P_{1}\left(r\right)\left[1+3\cos\left(2\phi \right)\right], \end{array}$$

where the displacement vector components are $\vec{U}$ = (*u_r_*, *u_φ_*, *u_θ_*).

Similarly, solution of (1) under the assumption of cylindrical symmetry (2D case) can be found in the following form:

(3) $$\begin{array}{c} \begin{array}{ccc} u_{r}=V_{r}(r)\cos(2\phi ), & u_{\phi }=V_{\phi }(r)\sin(2\phi ), & u_{z}=0, \end{array}\\ p=P_{0}(r)+P_{1}(r)\cos(2\phi ), \end{array}$$

where the components of the displacement vector are $\vec{U}$ = (*u_r_*, *u_φ_*, *u_z_*).

Using the incompressibility condition, the relationships between the *V_r_*(*r*) and *V_φ_*(*r*) are

(4) $$\begin{array}{c} V_{\phi }=-\frac{1}{4}\left(2V_{r}+r\frac{\partial V_{r}}{\partial r}\right),\;\;\;\;\text{for}\;3\text{D},\\ V_{\phi }=-\frac{1}{2}\left(V_{r}+r\frac{\partial V_{r}}{\partial r}\right),\;\;\;\;\text{for}\;2\text{D}. \end{array}$$

Substituting expressions (2)–(4) into (1) and eliminating the pressure, we find *V_r_*(*r*):

(5) $$\begin{array}{c} V_{r}=c_{1}^{i}r+c_{2}^{i}r^{3}+c_{3}^{i}r^{-2}+c_{4}^{i}r^{-4},\;\;\;\;\text{for}\;3\text{D},\\ V_{r}=c_{1}^{i}r+c_{2}^{i}r^{3}+c_{3}^{i}r^{-1}+c_{4}^{i}r^{-3},\;\;\;\;\text{for}\;3\text{D}. \end{array}$$

Arbitrary constants *c*_1_^*i*^, *c*_2_^*i*^, *c*_3_^*i*^, *c*_4_^*i*^ vary for each [*r*_*i*_, *r*_*i* + 1_] layer. These unknown constants can be found using boundary conditions and the stress and displacement continuity conditions at the boundaries of each layer. To satisfy boundary conditions for (1), the displacements *u_r_* and *u_φ_* must be zero at *r* = 0 (the solution is limited at the center of the system of coordinates), and must match the strains applied at infinity. For uniaxial loading, the condition

(6) $$\underset{r\to \infty }{\lim}\frac{V_{r}\left(r\right)}{r}=\varepsilon_{0}$$

must be satisfied, where *ε*_0_ is axial strain at infinity. For homogeneous media, the solution of spherical and cylindrical problems (5) is the same linear function *V_r_*(*r*) = *ε*_0⋅_ *r*.

Hence, the solution of (1) for a specific elasticity distribution can be reduced to the solution of a linear system of algebraic equations. This fact allows us to simplify and to speed up the solution of the forward problem and, as a result, to construct an effective procedure for inverse problem solution.

### 2.2. Inverse problem

**Figure 2:**
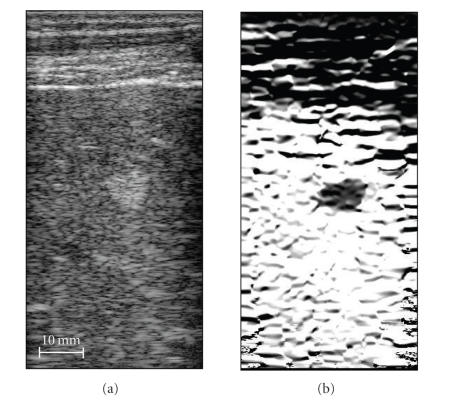
Liver hemangioma (a) B-Scan (left) and (b) strain image (right). The images are 38-mm by 78-mm.

The inverse problem was formulated using a Cartesian system of coordinates since a linear array ultrasound probe was used to measure the internal displacements and the ultrasound images were inherently obtained in a Cartesian system of coordinates (*x*, *y*, *z*) as evident from the ultrasound and strain images presented in Figure 2. Therefore, the analytical solutions of the forward problems formulated in spherical and cylindrical systems of coordinates were converted to a Cartesian system of coordinates. By pushing the ultrasound transducer against the skin, the external surface deformations we applied were such that the ultrasound beam is along the direction of applied deformation.

In ultrasound strain imaging, the quality of axial strain estimates (i.e., signal-to-noise ratio and resolution) is higher than that of other strain tensor components [19, 20]. Therefore, it is desired to construct the inverse problem solution using only one experimentally measured axial component of the strain tensor *ɛ_yy_* = *∂u_y_*/*∂ y*, where *u_y_* is the axial component of the displacement vector in Cartesian coordinates.

The theoretical distribution of axial strain in the region of interest can be computed when the following parameters are known: Young's moduli over a set of layers, the center of the inhomogeneity (*x*_0_, *y*_0_), and the effective deformation *ɛ*_0_ at infinity. Using the analytic solution of the forward problem and having experimentally measured the axial strain component, the unknown Young's modulus *E_i_* can be estimated by minimizing the error function, that is, the difference between experimentally measured and theoretically predicted axial strains [22]:

(7) $$\delta =\left\|\varepsilon_{yy}^{\exp}-\varepsilon_{yy}^{\text{theory}}\left(E_{i},\varepsilon_{0},x_{0},y_{0}\right)\right\|.$$

In general, the deformation *ɛ*_0_ and the center (*x*_0_, *y*_0_) of the object can be considered unknown and estimated simultaneously with the unknown distribution of the Young's modulus *E_i_* by minimizing the error function. Alternatively, the applied deformation *ɛ*_0_ and the center (*x*_0_, *y*_0_) of the object can be derived based on some a priori information.

Hence, elasticity reconstruction reduces to a minimization of the error function of (7) with respect to the unknown elasticity distribution, the geometry of the object, and the details of external loading.

To minimize (7), a gradient-based iterative procedure was implemented [22, 26]. The step size of the gradient method was chosen based on three estimates of error function *δ* The minimum of the error function was locally predicted using a second-order polynomial approximation of *δ* as a function of the unknown parameters under the restriction of a decreasing error. Then, a global linear prediction for all unknowns was used simultaneously to increase the rate of convergence, that is, iterative step sizes for the gradient method were chosen taking into account the second-order polynomial approximation of *δ* as a function of unknown parameters. This iterative process does not require any additional derivatives of displacement or strain and, therefore, compared to direct elasticity reconstruction methods, does not introduce additional noise associated with higher-order derivatives of the displacement vector and strain tensor components. The high computational speed is achieved by using the analytical solution to calculate forward problem with only a small number of unknown parameters.

## 3. ELASTICITY RECONSTRUCTION OF LIVER HEMANGIOMA

Hemangioma is the most prevalent benign tumor of the liver, occurring in up to 70% of the population. Hemangiomas can vary in size and be as large as several centimeters. These tumors are filled with vascular channels of various sizes and may also contain fibrous tissue. Thrombi (clotted blood) may be present in the vascular channels. Histologically, the hemangioma is characterized by large, thin-walled blood vessels completely filled with blood [27].

These asymptomatic lesions are often found incidentally on ultrasound or CT when imaging studies are undertaken for other reasons. Once diagnosed, no treatment is necessary, and only large, symptomatic hemangiomata are treated by surgical resection. The diagnosis of hemangioma, however, requires special imaging studies such as nuclear medicine scans using radioactive technicium tagged red blood cells, magnetic resonance or dynamic CT scans with contrast.

Liver hemangiomas can be clearly identified in the ultrasound B-Scan image as a hyperechoic region, and the margins of the tumor are usually well defined. However, routine ultrasound is suggestive but usually not diagnostic. Many other tumors in liver, some of which are malignant, may appear similar on the B-Scan. Therefore, there is a need to specifically diagnose a detected liver mass in the least invasive and most time/cost efficient way available.

In the literature, hemangiomas are often referred to as soft lesions filled with blood [27]. Therefore, elasticity imaging may help in the diagnosis of hemangiomas. Indeed, most solid tumors are usually harder than the background, so hemangiomas may be distinguished from other liver tumors based on their mechanical properties.

To test the hypothesis that elasticity imaging can detect and diagnose hemangiomas, studies on volunteers with previously diagnosed hemangioma were performed. All subjects gave informed consent, and this study was approved by the University of Michigan Institutional Review Board. In these experiments, the liver was imaged between the ribs using an Ultramark-9 ATL scanner with a linear 128-element array transducer operating at 5 MHz. The system was interfaced with a custom-made circuit board to acquire approximately 120 frames of real-time digital RF signals during 4 seconds. Within this interval, the array, attached to a deformational device residing on a clinical trolley (gurney), was pressed against the body to produce a modest deformation of the liver. In most experiments, surface deformations did not exceed 10–12 mm, and all volunteers indicated no discomfort from the applied stress.

In all experiments, frame-to-frame motion was estimated using a two-dimensional correlation-based phase-sensitive speckle tracking technique [28]. The 2D displacement was estimated from the position of the maximum correlation coefficient, where the axial displacement estimate was refined using the position of the phase zero crossing of the analytic signal correlation. Displacement error was further reduced by filtering spatially adjacent correlation functions prior to displacement estimation [28].

The ultrasound B-Scan image of a liver hemangioma is presented in Figure 2(a). This image is a typical example of hemangiomas, where the location, margins, and size of the tumor are clearly identified. Furthermore, the muscle layers can be easily recognized at the top. This and other images in Figure 2 are 38-mm by 78-mm, where the transducer is located at the top of the image.

The distribution of the normal axial strain (*ɛ_yy_*) is shown in Figure 2(b). This quantitative grayscale image is displayed from 0 to 10 percent strain, where full black corresponds to no strain and full white to 10% strain. The tumor is clearly visible as a low strain region indicating that overall it is harder than the background tissue.

Similar results were obtained from several other volunteers. The apparent overall hardness of a hemangioma, inferred from the strain image, is unexpected given the soft interior of the tumor. However, this result is consistent with information gathered by surgeons in the operating room—when large, symptomatic hemangiomata are treated by surgical resection, the intact hemangiomas are felt as hard lesions. Pressure applied to the hemangioma, however, ruptures it, releasing blood as it collapses. Therefore, a hemangioma feels hard even it is filled with blood, which has no or low-shear elasticity.

In general, a soft, fluid-filled sack can appear hard if it is encapsulated by a very hard, thin membrane. If the mechanical properties of the shell are similar to that of the lesion, the shell itself would not affect the strain pattern. If the shell is harder than the lesion, however, the strain magnitude inside of the lesion is reduced. In fact, for an infinitely hard and absolutely noncompliant shell, the strain inside of the lesion vanishes regardless of the internal material properties. Therefore, it is reasonable to assume that the thin membrane encapsulating a hemangioma is significantly harder than the lesion's core, and dominates the overall strain pattern within the tumor. The mechanical properties of the shell surrounding a lesion can significantly impact the strain distribution. In particular, the strain images of a heterogeneous lesion surrounded by a hard shell and a uniform hard inclusion appear very similar. It may be possible, however, to estimate the lesion composition in both cases using reconstructive elasticity imaging.

For elasticity imaging of hemangiomas, the mapping of Young's modulus was performed using two approaches: direct reconstruction and model-based reconstruction. Direct reconstruction numerically solves the discretized equilibrium equations for a plane strain condition [10]. The plane strain condition is a reasonable approximation for elasticity imaging of the liver, where deformations are applied through the rib cage resulting in negligible out-of-plane strains. This method does not require any a priori knowledge of the object, and no other assumptions are made. After defining a region of interest containing the lesion, the Young's modulus distribution is reconstructed relative to the modulus of the background tissue (i.e., liver).

In model-based approach we assume that the hemangioma can be modeled as a spherical object such that the elastic modulus within the imaging plane is simply a function of radial position from the center of the tumor. For a realistic tumor, this is a reasonable approximation if the tumor is near no external boundaries. Nevertheless, by assuming a simple model such as this, the model-based reconstruction in the vicinity of the tumor core is far less susceptible to strain noise compared to direct elasticity reconstruction.

**Figure 3:**
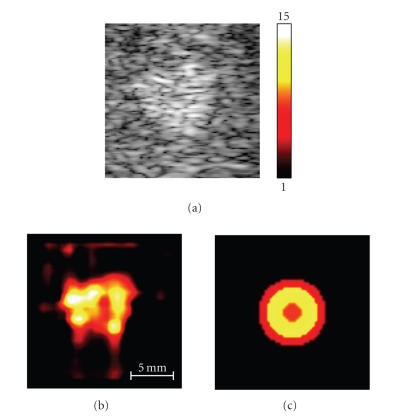
B-Scan (a) and elasticity images of hemangioma obtained direct method (b) and model-based method (c).

The results of the elasticity reconstruction are presented in Figure 3. In Figure 3(a), a 17.5-mm by 17.5-mm region of interest (ROI) of grayscale ultrasound image containing the hemangioma is shown. In direct reconstruction method (see Figure 3(b)), the Young's modulus along the ROI boundary was set to unity, resulting in reconstruction of the Young's modulus relative to liver. Clearly, the overall hemangioma is harder than the background tissue, but it has a softer interior part. This distribution is better depicted in the model-based elasticity image (see Figure 3(c)), where the softer interior part can be easily identified. In model-based reconstruction, the hemangioma was initially modeled as a homogeneous spherical inclusion, that is, object with one layer only. For a given number of layers, the relative Young's modulus, the external load *ɛ*_0_ and position of hemangioma center (*x*_0_, *y*_0_) were reconstructed by minimizing the error function (7) across the ROI.

**Figure 4:**
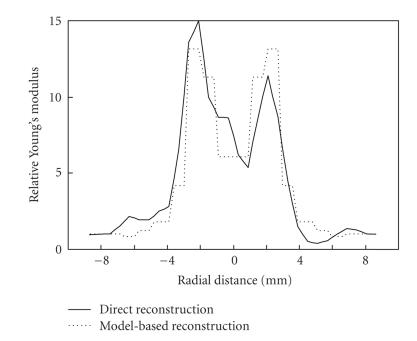
The Young's modulus distributions, obtained using direct and model-based reconstruction methods, are compared along the horizontal line intersecting the center of the hemangioma.

The Young's modulus distributions along the horizontal line intersecting the center of the hemangioma are contrasted in Figure 4 indicating reasonable agreement between the two different reconstruction approaches. In model-based reconstruction approach, only 12 layers were used to describe hemangioma. The results in Figures 3 and 4 correspond closely to the expected elasticity distribution within the hemangioma, where the capsule surrounding the tumor makes the lesion harder overall. Reconstructive elasticity imaging captures the complex composition of such tumors.

The results of this study suggest that diagnosis of liver hemangioma may be possible with reconstructive elasticity imaging. Strain imaging by itself may not be sufficient to differentiate hemangioma from other types of liver tumors since all lesions overall can produce somewhat similar strain images. In contrast, the reconstructed elasticity map may capture the critical differences between the tumors.

## 4. ELASTICITY RECONSTRUCTION OF DEEP VEIN THROMBOSIS

The leading cause of preventable in-hospital mortality in the USA and other developed countries is pulmonary embolism (PE), which is one of the complications of deep venous thrombosis [29]. DVT occurs when the blood clot forms inside a deep vein (commonly located in the calf or thigh) and either partially or completely blocks the flow of blood in the vein. In pulmonary embolism, a portion of the thrombus detaches from the vessel wall and travels through the veins into the lung. When large emboli lodge in the main pulmonary artery, pulmonary embolism can quickly become fatal. The level of pulmonary embolism risk and DVT treatment depend on the age of the clot (in this paper, we often refer to DVT as blood clot). For an acute thrombus, the patient is at a higher risk of the clot breaking off and becoming an embolus. This patient is treated with heparin followed by oral anticoagulants. Patients with chronic DVT are treated with either oral anticoagulants, coumadin, alone, or no treatment [30]. Because the risk of bleeding is higher with heparin than with coumadin, one would like to avoid the use of heparin if at all possible [31]. Therefore it is clinically important to distinguish between acute and chronic DVT.

Both magnetic resonance imaging (MRI) and duplex ultrasound can tell whether a thrombus is present [32, 33]. The problem is that while these technologies can identify the blood clot in the vein, they cannot determine its age. Studies suggest that elastic properties of clot can be used to determine DVT maturity [22, 34–41]. This is based on the assumption that the Young's modulus of a thrombus changes monotonically with fibrin and collagen concentration. Since both the fibrin and collagen content of a thrombus increase over time, DVT hardens with age. Consequently, remote estimation of the elastic properties of a thrombus can become an important clinical tool to age DVT.

The examination employs real-time B-mode sonography (2D grayscale ultrasound imaging) combined with color flow Doppler imaging and compression ultrasound. During the compression ultrasound, a transverse view of the veins and arteries of the patient's leg is imaged. The operator periodically pushes on the surface of the leg to deform the underlying tissue including vein and artery. If the vein does not deform while the adjacent artery does, the clot is suspected [33]. Since compression ultrasound already has all of the essential ingredients of elasticity imaging (i.e., external deformation of the object during continuous ultrasound imaging), elasticity imaging is a simple addition to the existing procedure of DVT diagnosis [36].

Animal studies were performed using a rat model of stasis-induced-venous thrombosis [37, 38, 42, 43]. The protocol was approved by the University of Michigan Committee on the use and care of animals and strictly complied with the National Institutes of Health Guide for the care and use of laboratory animals. A group of five rats were used in this study although only four animals developed thrombi. On the first day, all rats underwent surgery to initiate thrombus formation in the inferior vena cava (IVC). As the animals developed thrombi, which changed progressively from acute to chronic stages [42], each rat was imaged on days 3, 4, 5, 6, 7, 8, and 10.

All experiments were performed using a Siemens “Elegra” ultrasound scanner with a linear 12 MHz array transducer (VFX13-5). First, the IVCs were scanned in transverse and longitudinal orientations using color Doppler mode to determine thrombus location and find the best possible probe position on the rat's abdomen. Next, the transducer itself was used to compress the rat's abdominal wall and underlying tissue in a transverse orientation. It was attached to a manual deformation device to achieve continuous compression. The deformation lasted approximately 6 seconds, while phase sensitive ultrasound frames were collected in real time. Consecutive frames were processed offline using a 2D correlation-based phase-sensitive speckle tracking algorithm to derive the strain image of the DVT and surrounding tissue [28]. A kernel size 0.60-mm laterally by 0.17-mm axially was chosen for cross-correlation. The correlation function was filtered by a Hanning filter extending 1.20-mm laterally by 0.85-mm axially. Finally, frame-to-frame displacements were accumulated over a number of frames within the same deformation sequence and axial strains were calculated numerically.

**Figure 5:**
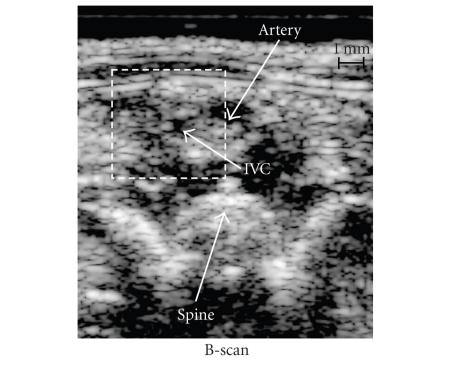
Ultrasound B-scan image of rat's abdomen.

**Figure 6:**
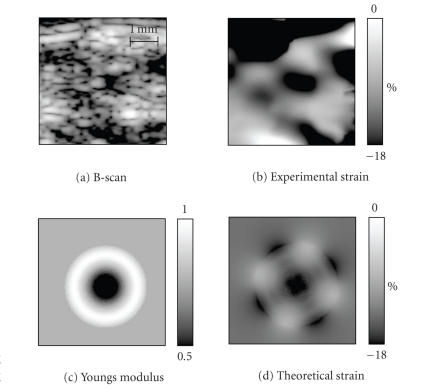
Elasticity reconstruction procedure demonstrated for a rat with a 5-day old thrombus. (a) A 4.5-mm by 4.5-mm region of interest (a) was selected for elasticity reconstruction. (b) The measured axial strain image within selected ROI. (c) Reconstructed Young's modulus distribution. (d) Corresponding theoretical axial strain image.

An analysis of the B-scan in the thrombus area (see Figure 5) was the starting point of elasticity reconstruction for each animal. Based on the ultrasound image, the ROIs for elasticity reconstruction and the position of the clot center (*x*_0_, *y*_0_) were chosen. Each ROI was selected such that it included the IVC and a small portion of surrounding tissue (see Figure 6) to minimize elasticity variations of background tissue. The elasticity reconstruction program creates relative Young's modulus images—the stress distribution at the surface in response to surface-applied deformations is required for absolute reconstruction. Therefore, a vessel wall was used as the reference material, that is, Young's modulus of the vessel wall was assigned unity value.

A slight modification of described method was used for DVT elasticity reconstruction. We assume that the external load is applied at an angle *α* relative to the axial direction of an ultrasound beam. The angle *α* was added to expression (7) as a parameter of minimization. The details are presented elsewhere [22]. Based on the geometry of the thrombus, vessel, and surrounding tissue (Figure 1), the clot-containing vessel is modeled as a long round cylinder. At the beginning of the reconstruction, a homogeneous cylindrical clot was assumed. The position of the clot center (*x*_0_, *y*_0_) was manually set based on the ultrasound image.

Once the solution for a given number of rings was found, the number of rings required to represent the object was increased. The previous elasticity distribution and load parameters *α* and *ɛ*_0_ were used (after interpolation) as the initial point for the next step, where thrombus, vessel wall, and surrounding tissue were approximated using more independent rings of different elasticity. This iterative process was continued until the difference between two successive reconstructions (i.e., Young's modulus reconstructions for a different number of layers) was less then 2%. In the final Young's modulus map, the number of layers ranged from 30 to 40 depending on a particular dataset.

A typical B-scan image of a rat's abdomen with 5-day old thrombus is shown in Figure 5. This image covers 10-mm (axial) by 8.5-mm (lateral) region. Using color-flow imaging, the artery adjacent to the IVC was identified while no flow was detected in the IVC signaling that the thrombus occluded the vessel. On the bottom of the B-scan, the anterior surface of the spine can also be noted. Figure 6 presents the results of model-based elasticity reconstruction for the same rat. The 4.5-mm by 4.5-mm elasticity reconstruction region (Figure 6(a), also outlined rectangular area in Figure 5) was chosen to include the entire IVC, with the clot located in the center, and a small portion of background tissue. The measured axial strain image is presented in Figure 6(b). This image is displayed over a 0 to 18% strain dynamic range, where full white represents no strain, and full black represents a normal axial strain magnitude of 18% and larger (negative strain indicates that vein size was reduced vertically during the deformation).

Once the region of interest was identified, iterative elasticity reconstruction was performed, where the experimentally measured axial strain distribution *ɛ_yy_* ^exp^ was compared with the theoretically predicted axial strain map *ɛ_yy_* ^theory^ to minimize the error function (7). The resultant Young's modulus distribution * E_i_* over a set of rings is presented in Figure 6(c) where a grayscale map is used to display the relative Young's modulus over the 0.5 to 1.0 range. The grayscale map was selected so that full black corresponds to a relative Young's modulus of less than or equal to 0.5, and white areas represent harder tissue. Finally, Figure 6(d) presents the theoretically predicted axial strain distribution corresponding to reconstructed values of Young's modulus in Figure 6(c).

**Figure 7:**
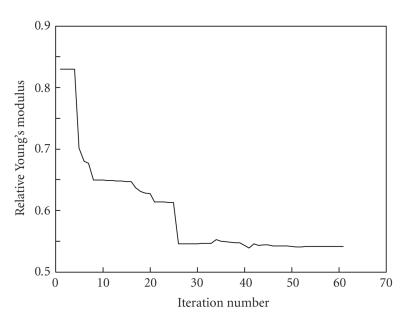
Relative Young's modulus versus number of iterations for blood clot approximated using 5 rings or layers. Initial value for iterative process was chosen from previous 4-ring model. These particular results correspond to the data presented in Figures 5 and 6 (an animal with 5-day old thrombus).

**Figure 8:**
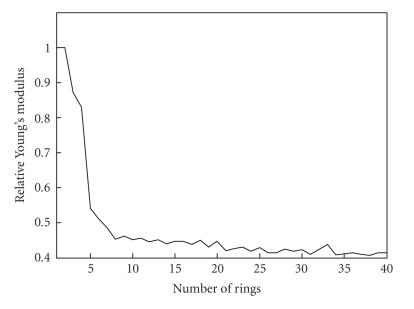
Changes in relative Young's modulus with increased number of layers or rings. These particular results correspond to the data presented in Figures 5 and 6 (an animal with 5-day old thrombus).

The minimization process is illustrated in Figures 7 and 8. Figure 7 shows changes in relative Young's modulus during iterations. In this example, the number of layers is fixed and equal to 5. The initial elasticity value was taken from the previous step, where the number of layers was equal to 4. During each iteration, the minimization of the error function (7) was performed. Regions of rapid change in elasticity (at iterations 5 and 25) correspond to successful global linear prediction resulting in 5–10 times faster convergence. Figure 8 presents behavior of relative elasticity of thrombus as the number of rings is increased. These results illustrate that 30–40 rings are sufficient to describe the blood clot, vessel wall, and surrounding tissue for this particular experimental dataset since further increase in the number of rings does not change the final result.

**Figure 9:**
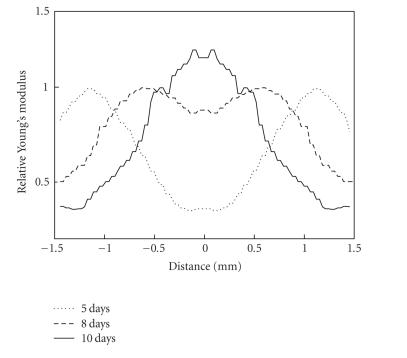
Young's modulus profiles for 5-day old (acute), 8-day old (subacute), and 10-day old (chronic) thrombi.

**Figure 10:**
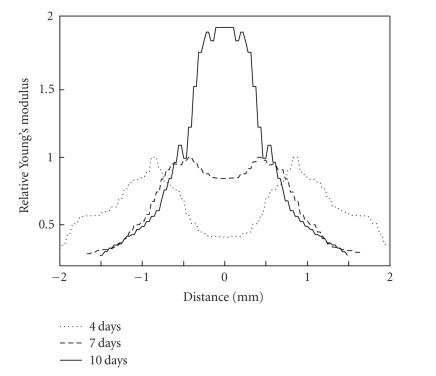
Young's modulus profiles for 4-day old (acute), 7-day old (subacute), and 10-day old (chronic) thrombi.

Figure 9 contrasts relative Young's modulus profiles along the center of the thrombus for one of the animals at 5, 8, and 10 days after the surgery (the profile for 5-day old thrombus corresponds to results presented in Figures 5–8). Figure 10 presents the profiles of relative Young's modulus for another animal with 4-day, 7-day, and 10-day old thrombus. Note here, that position of vessel walls have changed through time because of the vessel shrank in process of clot formation [37, 38].

**Figure 11:**
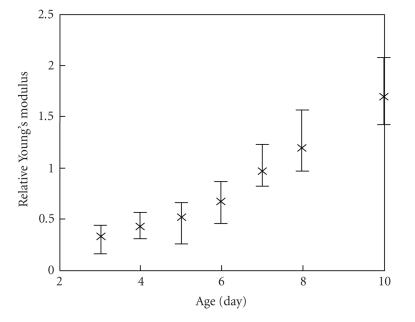
The model-based reconstructed values of relative Young's modulus of the blood clot during formation and aging of thrombus.

In this animal model, 3-day to 4-day old thrombus represents an acute DVT, 6-day to 7-day old thrombus represents a subacute DVT, and 10-day old thrombus represents a chronic DVT. The rate of thrombus growth is different in humans, but the process of clot formation has the same stages. As the thrombus develops, its elasticity increases. This is further illustrated in Figure 11, where relative Young's modulus is plotted as function of time after surgery, that is, age of the thrombus. Here, the elasticity values were averaged for the four rats used in our study. Clearly, the relative elasticity of the clot increases with thrombus age suggesting that Young's modulus can be used to age DVT.

**Figure 12:**
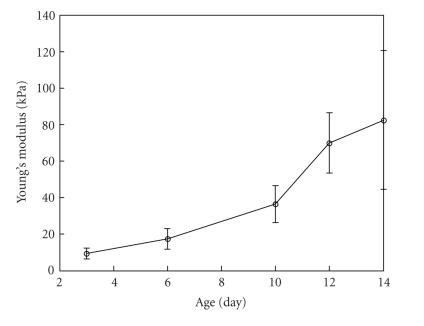
The ex vivo direct mechanical measurements of the Young's modulus of the blood clot during formation and aging of thrombus.

The results of in vivo elasticity reconstruction were compared with the results of ex vivo direct elasticity measurements [38]. A total of 59 Sprague-Dawley rats were studied. On days 3, 6, 10, 12, and 14, the group of animals (9–13 animals) was euthanized for the direct measurements of Young's modulus of the blood clots. Thrombi were removed from the dissected IVC and transported immediately to the next room for mechanical load-displacement measurements to accurately assess the Young's modulus of the thrombi. Specifically, the displacement versus force was measured while a test sample was subjected to compression. Based on the strain-stress relationship and finite element modeling of the clot deformation, Young's modulus of the test sample was estimated. Initially, the system was tested on rubber cylindrical sample with known elastic properties [38]. The direct ex vivo elasticity measurements were performed using 15–20% strain range. This range approximately corresponds to the strain level used in ultrasound in vivo studies. In Figure 12, the mechanical, ex vivo measurements of Young's modulus are plotted as function of thrombus age.

Figures 11 and 12 demonstrate that the in vivo reconstruction agrees well with the ex vivo mechanical measurement. The results of direct elasticity estimations and remote assessment of the clot elasticity indicate that a thrombus hardens as it matures. Indeed, the Young's modulus of thrombi increases over time, and a 10-day old thrombus is approximately 3–6 times harder than a 3-day old thrombus.

Therefore, the model-based elasticity reconstruction approach is applicable to an animal model of DVT. Indeed, thrombus elasticity (Young's modulus) increases with time and can be accurately assessed and monitored using quantitative model-based reconstructive elasticity imaging.

## 5. CONCLUSIONS

The model-based elasticity reconstruction method was developed to demonstrate the potential of ultrasound-based reconstructive elasticity imaging. The developed model-based method has several advantages compared to other elasticity reconstruction approaches. The model-based elasticity reconstruction requires only axial component of the strain tensor. The minimization procedure in model-based reconstruction is relatively efficient and stable given the small number of unknown scalar parameters derived from an analytic solution of the forward elastic problem. Finally, the model-based reconstruction is not highly sensitive to the details of external loading. The model-based reconstructive elasticity imaging was applied to differentiate liver hemangioma and to age deep vein thrombosis. The results of our studies indicate that the model-based reconstruction approach is applicable in ultrasound in vivo elasticity imaging and can provide needed information about biomechanical properties of tissues.
